# Non-functional bladder paraganglioma in a young patient: conservative management by transurethral resection and diagnostic challenges

**DOI:** 10.11604/pamj.2025.52.105.48105

**Published:** 2025-11-12

**Authors:** Ghassane El Omri, Omar Iraqui Houssaini, Moussaab Rachid, Younes Houry, Abdeljalil Heddat

**Affiliations:** 1 Department of Urology, Cheikh Khalifa International University Hospital, Mohammed VI University of Sciences and Health, Casablanca, Morocco

**Keywords:** Paraganglioma, urinary bladder neoplasms, transurethral resection of bladder, case report

## Abstract

Bladder paraganglioma is a rare neuroendocrine tumor, accounting for less than 0.05% of all bladder tumours. Its clinical presentation is often variable, making diagnosis challenging, particularly in non-functional forms. We report the case of a patient with a bladder paraganglioma revealed by recurrent haematuria, in the absence of clinical signs of catecholamine secretion. Transurethral resection enabled complete treatment, although the procedure was complicated by stimulation of the obturator nerve, without a notable incident. Postoperative investigations, including follow-up cystoscopy and urinary metanephrine levels, were unremarkable. This case illustrates the diagnostic challenges associated with non-secretory forms. The diagnosis was confirmed by histological and immunohistochemical analysis, which ruled out urothelial carcinoma. Treatment is based on complete resection, and prolonged surveillance is warranted due to the rare but real risk of recurrence or malignant transformation. Although rare, bladder paraganglioma should be considered in the presence of any atypical bladder mass. Appropriate management and close follow-up are essential to ensure an excellent prognosis.

## Introduction

Paragangliomas are rare neuroendocrine tumors arising from extra-adrenal chromaffin cells. When located in the bladder, they account for less than 0.05% of all bladder tumors and approximately 79% of paragangliomas of the urogenital tract [1]. Functionally, these tumors may secrete catecholamines, leading to characteristic symptoms such as paroxysmal hypertension, headaches, palpitations, or micturition-induced syncope [2]. However, a significant proportion remain non-functional and present with nonspecific urological symptoms, most commonly isolated episodes of hematuria [3]. The deceptive clinical presentation of this tumor can make diagnosis challenging for both the clinician and the pathologist. The latter plays a key role in confirming the diagnosis, particularly in non-secreting forms that may mimic poorly differentiated urothelial carcinomas [4]. Moreover, unawareness of the tumor´s functional nature exposes the patient to a high risk of hemodynamic complications during diagnostic or surgical procedures [2]. A precise preoperative diagnosis is therefore crucial to ensure safe and appropriate management. We report here a case of non-functional bladder paraganglioma revealed by recurrent hematuria in a young patient, highlighting the diagnostic and therapeutic challenges associated with this rare entity.

## Patient and observation

**Patient information:** a 19-year-old male patient with no significant past medical history presented with multiple episodes of macroscopic hematuria evolving over a six-week period. The hematuria was terminal in nature and associated with clot formation. It was accompanied by irritative lower urinary tract symptoms (pollakiuria and urgency), without associated pain or fever.

**Clinical findings:** on clinical assessment, there were no symptoms suggestive of catecholaminergic secretion, such as paroxysmal hypertension, palpitations, profuse sweating, or headaches. There was no reported family history of pheochromocytoma or paraganglioma. Physical examination was unremarkable, with normal blood pressure (125/78 mmHg) and a regular heart rate. Abdominal examination did not reveal any palpable mass, and the genitourinary examination was within normal limits.

**Figure 1:**
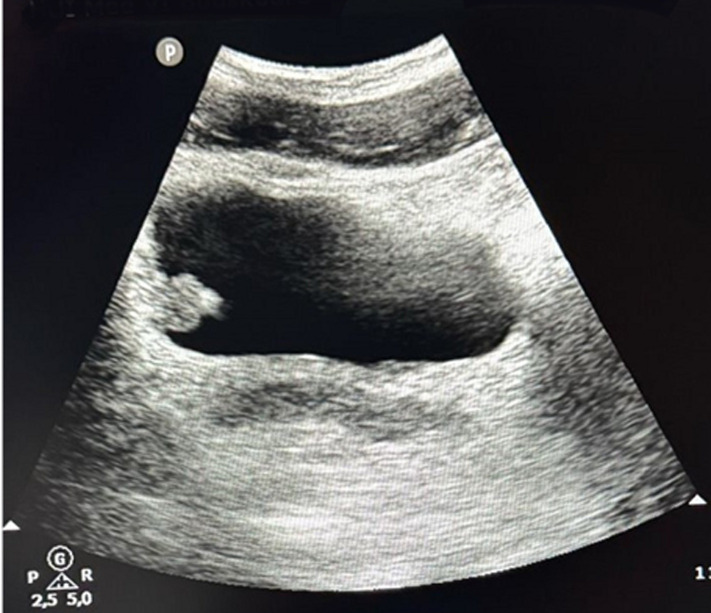
a bladder ultrasound revealed a polypoid mass arising from the right lateral wall

**Figure 2:**
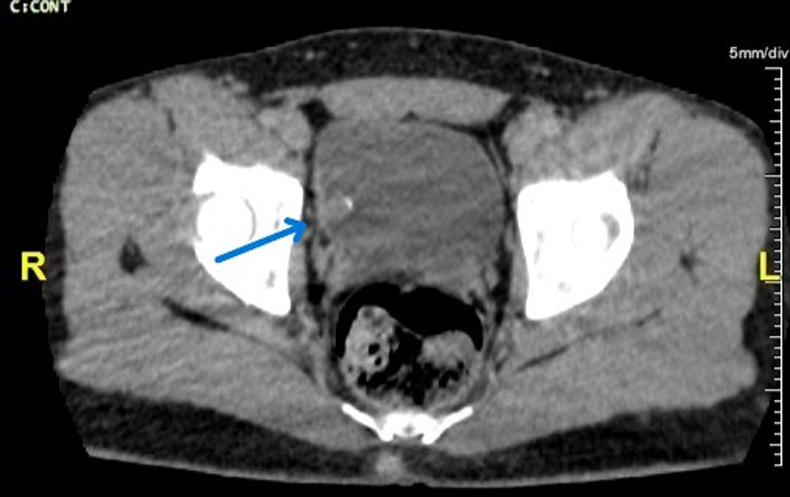
computed tomography scan confirming a hypervascular soft tissue lesion without locoregional extension or impact on the upper urinary tract

**Diagnostic assessment:** initial laboratory investigations, including complete blood count, serum creatinine, and urinalysis, were unremarkable, apart from confirmation of hematuria. A bladder ultrasound revealed a 2.5 cm polypoid intravesical tissue mass arising from the right lateral wall of the bladder, with no signs of local invasion (Figure 1). A computed tomography (CT) confirmed the presence of this hypervascular lesion, with no abnormalities identified in the upper urinary tract (Figure 2).

**Figure 3:**
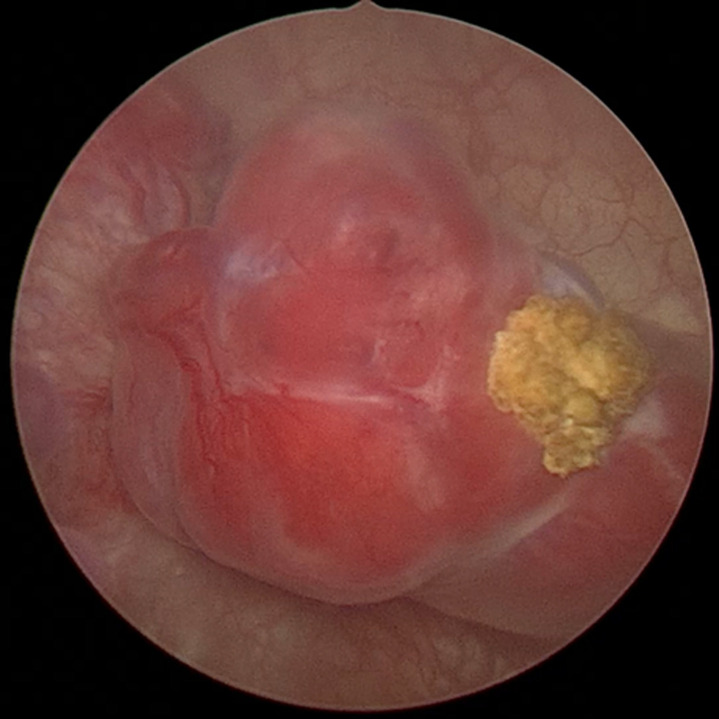
endoscopic appearance of the tumor lesion in the bladder

**Therapeutic interventions:** in light of the ultrasound and CT findings suggestive of a bladder tumor, a transurethral resection of the bladder (TURB) was indicated. The procedure was performed under locoregional anesthesia without any intraoperative complications. A complete resection of the lesion was achieved, respecting anatomical planes and including deep chips extending to the detrusor muscle to allow for accurate histopathological staging. Intraoperatively, the tumor appeared macroscopically polypoid and highly vascular, with a narrow base of implantation (Figure 3). It is worth noting that resection was repeatedly hindered by stimulation of the obturator nerve, leading to reflex adductor muscle contractions. However, no intraoperative complications such as bladder perforation or significant hemorrhage occurred. No hypertensive episodes or arrhythmias were observed during manipulation of the tumor.

**Figure 4:**
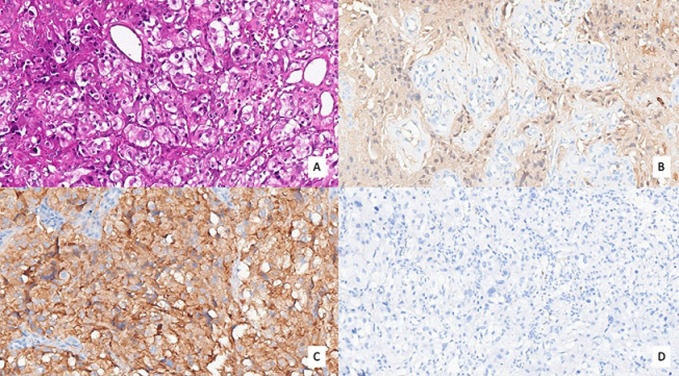
histopathological and immune histochemical features of bladder paraganglioma; A) H&E staining showing a nodular proliferation of tumor cells arranged in nests, separated by a fine vascular network; B) S-100 immunohistochemistry: staining of sustentacular cells at the periphery of the tumor nests; C) synaptophysin immunohistochemistry: diffuse cytoplasmic staining of tumor cells, confirming their neuroendocrine nature; D) CK7 immunohistochemistry: absence of expression in tumor cells, helping to rule out urothelial carcinoma

**Follow-up and outcome of interventions:** a bladder catheter was left in place for 48 hours, with continuous bladder irrigation initiated prophylactically during the immediate postoperative period. The postoperative course was uneventful, with no fever or secondary hematuria. The patient was discharged on postoperative day 3, after recovery of bowel function and satisfactory spontaneous voiding. Macroscopic examination of the resected fragments revealed multiple friable tumor pieces, ranging in color from brownish to reddish. Histologically, the tumor was composed of dense nests of cells separated by a delicate vascular network. The tumor cells exhibited abundant eosinophilic cytoplasm and round to oval nuclei, sometimes hyperchromatic, without marked atypia or significant mitotic activity. The stroma appeared richly vascularized (Figure 4 A). This morphological pattern suggested two main differential diagnoses: a bladder paraganglioma or an infiltrating urothelial carcinoma. Immunohistochemical analysis guided the diagnosis, demonstrating strong expression of neuroendocrine markers (chromogranin A and synaptophysin), along with peripheral staining of sustentacular cells by S-100 protein (Figure 4B,C). The absence of urothelial marker expression (CK7, CK20, p63) excluded urothelial carcinoma, confirming the diagnosis of intravesical paraganglioma (Figure 4D). Following histological confirmation of bladder paraganglioma, a staging work-up was undertaken to assess for locoregional or distant disease.

**The following investigations were performed:** thoracic CT scan revealed no suspicious thoracic or mediastinal abnormalities. Pelvic MRI was not performed in the immediate postoperative period due to inflammatory changes related to the resection, which rendered morphological interpretation unreliable. Urinary levels of free methoxylated metabolites (metanephrines and normetanephrines), measured at a later stage, were within normal limits, supporting the diagnosis of a non-secreting paraganglioma. Follow-up cystoscopy, performed after bladder healing, showed a macroscopically normal bladder, with no residual lesion or evidence of recurrence. Iodine-123 metaiodobenzylguanidine 123 (MIBG) scintigraphy was not performed, given the absence of clinical or biochemical indications suggestive of residual or metastatic disease. To date, after close clinical follow-up, no recurrence has been observed. The non-invasive, non-secreting, and localized nature of the lesion confers a favorable short-term prognosis, provided regular surveillance is maintained.

**Patient perspective:** the patient was pleased with the care he received throughout therapy.

**Informed consent:** written informed consent was obtained from the patient for participation in our study.

## Discussion

Bladder paraganglioma is a rare neuroendocrine tumor, accounting for less than 0.05% of all bladder tumors and approximately 6% of extra-adrenal paragangliomas [1]. Arising from chromaffin cells of the autonomic nervous system, it typically develops in the submucosa of the bladder, most commonly in the trigone and lateral wall [5]. The majority of these tumors are functional and secrete catecholamines, resulting in a characteristic clinical presentation. However, non-functional forms, as in our case, make the diagnosis more challenging. Clinically, hematuria is the most frequent presenting symptom, observed in nearly 60% of cases [3]. Symptoms related to catecholamine hypersecretion (hypertensive crises, profuse sweating, palpitations, or even micturition-induced syncope) are present in 30-40% of cases [2], but may be absent in non-secreting forms. In such cases, the clinical picture may be limited to nonspecific lower urinary tract irritative symptoms [5], thereby delaying the diagnosis. Definitive diagnosis relies on histological examination combined with a targeted immunohistochemical profile. From a pathological standpoint, the primary challenge lies in distinguishing between bladder paraganglioma, a rare entity with a favorable prognosis, and infiltrating urothelial carcinoma with a nested growth pattern, which is far more common and aggressive [4]. These two entities share a nodular, minimally atypical architecture, especially at low magnification, which can easily lead to misdiagnosis [4]. In this context, immunohistochemistry is essential. The presence of cell nests separated by a delicate vascular network, suggestive of a Zellballen Pattern, pointed toward a paraganglioma but lacked specificity [4]. The diagnosis was confirmed by strong immunoreactivity for neuroendocrine markers (chromogranin A, synaptophysin), peripheral S-100 staining of sustentacular cells, and absence of expression of urothelial markers (CK7, CK20, p63), effectively ruling out urothelial carcinoma [4].

This case highlights the importance of considering this rare diagnosis when evaluating an atypical bladder mass, particularly in a young patient and in the absence of a classic functional presentation [4]. Magnetic resonance imaging (MRI) is generally the preferred modality for locoregional staging [6]. In our case, an MRI was not performed immediately after resection due to postoperative inflammatory changes that could compromise image interpretation. Furthermore, postoperative urinary levels of methoxylated metabolites were within normal limits, confirming the absence of functional secretion. More specifically, 123MIBG scintigraphy and Radiolabeled Gallium DOTA-1-NaI (3)-octreotide (68Ga-DOTANOC-PET) scans are primarily indicated in cases with suspected hormonal activity or for evaluating metastatic spread [7]. They also play a role in post-treatment surveillance by confirming the absence of residual tumor or distant metastases [8]. The gold standard treatment is complete tumor excision, which may be achieved endoscopically in the case of small, superficial lesions [1,9], or via partial cystectomy for larger, infiltrative tumors or when margins are involved [10]. In our case, a complete transurethral resection was performed. We encountered repeated reflex contractions of the adductor muscles due to obturator nerve stimulation, a well-known phenomenon during lateral wall resections, but no intraoperative complications occurred. In the presence of clinical signs suggestive of a secretory paraganglioma, specific preoperative preparation is essential. This includes initial alpha-adrenergic blockade (typically with phenoxybenzamine or doxazosin) to achieve blood pressure control, followed by beta-adrenergic blockade if tachycardia persists [7]. The prognosis of bladder paragangliomas is generally favorable following complete surgical excision. However, long-term follow-up is necessary due to the risk of local recurrence and the rare potential for metastatic progression. Predictive factors of malignancy are primarily clinical and evolutionary, as conventional histological criteria are not always reliable indicators of aggressive behavior [7].

## Conclusion

Our case highlights that, even in the absence of typical clinical manifestations, bladder paraganglioma should be considered in the differential diagnosis of non-urothelial bladder masses, particularly in young patients. A systematic hormonal assessment and appropriate preoperative preparation are essential to prevent intraoperative complications. Histological and immunohistochemical confirmation remains crucial, not only to establish the definitive diagnosis but also to avoid confusion with other, more common and potentially more aggressive bladder tumors. Long-term follow-up is recommended due to the risk of late recurrence.
